# Planning and production of grammatical and lexical verbs in multi-word messages

**DOI:** 10.1371/journal.pone.0186685

**Published:** 2017-11-01

**Authors:** Violaine Michel Lange, Maria Messerschmidt, Peter Harder, Hartwig Roman Siebner, Kasper Boye

**Affiliations:** 1 Department of Nordic Studies and Linguistics, Copenhagen University, Copenhagen, Denmark; 2 Danish Research Centre for Magnetic Resonance, Copenhagen University Hospital Hvidovre, Copenhagen, Denmark; 3 Department of English, Germanic and Romance Studies, Copenhagen University, Copenhagen, Denmark; 4 Department of Neurology, Copenhagen University Hospital Bispebjerg, Copenhagen, Denmark; Waseda University, JAPAN

## Abstract

Grammatical words represent the part of grammar that can be most directly contrasted with the lexicon. Aphasiological studies, linguistic theories and psycholinguistic studies suggest that their processing is operated at different stages in speech production. Models of sentence production propose that at the formulation stage, lexical words are processed at the *functional level* while grammatical words are processed at a later *positional level*. In this study we consider proposals made by linguistic theories and psycholinguistic models to derive two predictions for the processing of grammatical words compared to lexical words. First, based on the assumption that grammatical words are less crucial for communication and therefore paid less attention to, it is predicted that they show shorter articulation times and/or higher error rates than lexical words. Second, based on the assumption that grammatical words differ from lexical words in being dependent on a lexical host, it is hypothesized that the retrieval of a grammatical word has to be put on hold until its lexical host is available, and it is predicted that this is reflected in longer reaction times (RTs) for grammatical compared to lexical words. We investigated these predictions by comparing fully homonymous sentences with only a difference in verb status (grammatical vs. lexical) elicited by a specific context. We measured RTs, duration and accuracy rate. No difference in duration was observed. Longer RTs and a lower accuracy rate for grammatical words were reported, successfully reflecting grammatical word properties as defined by linguistic theories and psycholinguistic models. Importantly, this study provides insight into the span of encoding and grammatical encoding processes in speech production.

## General introduction

There is little experimentally based knowledge on multi-word production contrasting grammatical words (e.g. articles and auxiliaries) and lexical words (e.g. adjectives and full verbs). Existing models of speech production suggest that lexical elements are planned prior to grammatical ones, but these models are either based on analyses of errors in text corpora [1, 2] or on experimental studies of single-word production only [3, 4] Additionally, many studies are based on distinctions between “function” and “content words” or between “open-” and “closed-class words” which are not grounded in linguistic or psycholinguistic theory, which is problematic from a cross-linguistic view. The fact that studies comparing the two types of words are performed with rather artificial designs and not properly anchored theoretically makes it difficult to generalize models of speech production to spontaneous speech production. In this study, we address those methodological and theoretical issues and intend to shed light on the nature of the difference between grammatical and lexical words. We investigate whether grammatical and lexical verbs are planned and produced differently when fully controlled for potential psycholinguistic confounds and produced in a fairly natural context. Gaining insight into the mechanisms underlying the two types of words will help further investigation of some of the predictions made by linguistic and psycholinguistic models. The study was designed to test specific hypotheses that were derived from a novel usage-based theory of the grammar-lexicon distinction [5]. Predictions based on this theory were tested in an experiment where we compared the production of grammatical verbs (auxiliaries) with lexical verbs (full verbs) in perfectly matched homonymous sentences. Reaction times (RTs), articulation duration of the entire sentence and accuracy rates were measured. Differences between the production of grammatical and lexical verbs are discussed in the context of existing linguistic and psycholinguistic models with an emphasis on theory-based properties that distinguish grammatical from lexical words.

### Grammatical vs. lexical words—Theoretical challenges

The distinction between lexical and grammatical words draws on the distinction between lexicon and grammar from a linguistic perspective. Note that from this perspective, the lexicon represents lexical words (like nouns, verbs, adverbs) which is not to be confused with the so-called mental lexicon from a psycholinguistic perspective which contains all words (including grammatical ones). Grammar does not only comprise grammatical words, but also grammatical morphemes (affixes) and syntactic structures. From a psycholinguistic and experimental point of view, however, grammatical words are of particular interest as they constitute the aspect of grammar that can most directly be contrasted with the lexicon. Unlike affixes and syntactic structures, grammatical words share with lexical words the property of being words. In a sense the distinction between lexical and grammatical words meets a theoretical vacuum. The influential theory of Generative Grammar makes a distinction between lexicon and grammar [6], but with its focus on grammar it has little to say about lexical words. Generative Grammar also has little to say about grammatical words because grammar is conceptionalised as rules rather than items. The resulting lack of interest in the distinction between lexical and grammatical words is reflected in an influential theory of the cognitive underpinnings of the lexicon-grammar distinction: Ullman [7]relates lexicon to declarative memory and grammar to procedural memory. Like Generative Grammar, Ullman´s model focuses on the rules aspect of grammar; it remains agnostic when it comes to the question whether grammatical words are stored in declarative or in procedural memory. At the opposite pole of the landscape of linguistic theories, cognitive linguistics, especially Construction Grammar, downplays the distinction between lexicon and grammar (see [8]: 559 for a recent statement). According to Construction Grammar, all lexical and grammatical aspects of language are dealt with as items, and all items are taken to be stored in a common “constructicon”. This means that in Construction Grammar there is even less room for the distinction between lexical and grammatical words than in Generative Grammar.

As a result of this, the distinction between lexical and grammatical words is often defined independently of linguistic theory in terms of distinctions between “function words” (words without content) and “content words” (words with content) or between “closed-class words” (words which do not accept any new words in their category) and “open-class words” (words which accept new words in their category). Both these distinctions are highly problematic. As for the first one, a tense auxiliary like *will*, a preposition like *of* in *the wife of my friend* and articles like *the* clearly have a content (‘future’, ‘possession’, ‘definiteness’ respectively). In fact, lexical and grammatical words may have similar contents, as in the case of *have* in *my friend has a husband* and *of* in *the husband of my friend*, both of which express ‘possession’. Accordingly, the terms “content words” and “function words” are often used simply as convenient labels without reference to either content or function, and defined in terms of the second distinction between open-class and closed class words [9]. This second distinction is however no less problematic. What belongs to a closed class in one language may belong to an open class in another one. In some languages, for instance, verbs belong to closed classes [10]. Moreover, there are indications that at least some closed classes are heterogeneous. In a letter detection study, [11] found that prepositions clustered midway between grammatical words (complementizers and determiners) on the one hand, and lexical words (nouns, verbs, adjectives and adverbs) on the other. A possible account is that while all prepositions belong to a “closed class”, some are items belonging to the first, grammatical group while others are like items belonging to the second, lexical one. Studies with aphasic speakers also show that not all words are affected equally within their own category [12, 13]. If the mental lexicon could be divided into two clear-cut categories (content/function or closed/open class words), then speech pattern of aphasic speakers could easily be predictable based on this simple dichotomy. However, this is not the case as agrammatic speakers, for example, may still produce some “closed class” words. This pattern suggests that the proposed distinction might not be as straightforward as it seems.

In a psycholinguistic context, the problem with theory-independent distinctions such as “function” vs. “content words” or “open-class” vs. “closed-class words” is that no empirical predictions can be made due to the lack of a theoretical framework. Even if empirical measurements revealed differences in linguistic processing depending on these theory-independent labels, it would remain unclear what triggered such differences. It is not very plausible, for instance, that psycholinguistic differences between elements are triggered by their membership of “open” vs. “closed classes” of words, and there is also a risk that the pre-theoretical labels bias our understanding of the distinctions that are indeed psycholinguistically real. The lack of theoretical framework regarding the nature of the difference between grammatical and lexical word is important since we build studies based on those words in order to develop models of speech production. But if the contrasts we use in the first place are inaccurate (some words are classified as being one type when they might be classified as being another), then the models will contain a lot of noise.

### Theoretical background and hypotheses

The present study meets the theoretical challenges discussed above by anchoring the distinction between lexical and grammatical words in a novel theory of the grammar-lexicon distinction [5]and by exploiting this theory to derive grammar-specific and lexicon-specific hypotheses with respect to multi-word language production. According to this theory [5], grammatical items (morphemes, words, constructions) are ancillary in relation to lexical ones. This gives rise to two fundamental differences between grammatical and lexical items. The first difference involves discourse (or information) prominence. Grammatical items are conventionalized (= coded) and hence entrenched with secondary (or backgrounded) status. In contrast, lexical items have the potential to express the primary (or foregrounded) point of an utterance. This entails that they are entrenched with a higher degree of prominence than grammatical items. The second difference is caused by structural requirements. Grammatical items are necessarily dependent (one cannot say *a* or *-ed* in isolation); In contrast, at least some lexical elements may be produced or comprehended in isolation (one can say *run*! and still convey the intended meaning). In other words, grammatical words have two defining properties that distinguish them from lexical words: they always carry secondary information, and they are all dependent on other items. While distinct, these two properties are closely related: secondary status inevitably presupposes co-occurrence of a host with respect to which an item with secondary status can be secondary [5]. We shall refer to the first property of grammar as the *low prominence* property and to the other one as the *dependence* property. These two properties give rise to different, but compatible predictions concerning the behaviour of grammatical words in multi-word language production.

#### Low prominence of grammatical words and its implication for language production

Given the *low prominence* property of grammar, it can be predicted that in cases of resource limitations, grammatical words are omitted to a higher degree than lexical ones. This prediction is confirmed by instances of telegraphic speech phenomena in the context of telegram writing (where space or time resources are sparse) and possibly agrammatic aphasia (where cognitive resources may be sparse; e.g. [14].

In the present study, the experimental setting entailed non-restricted language production. In such a setting where resources are not limited, two different predictions can be made. First, a lower amount of articulatory energy might be invested in grammatical than in lexical words. This may be reflected in the tendency for grammatical items to be phonologically short. Second, grammatical words being less crucial for communicative purposes might be produced less accurately as they are paid less attention to. This might be reflected by a lower accuracy rate for grammatical words. Earlier studies have indeed shown a link between speech production errors and attention mechanisms [15,16]. We shall refer to these two predictions as the *prominence hypothesis*. Note that the second of these predictions is shared with the dependence hypothesis (see below).

#### Dependence of grammatical words and its implications for language production

The *dependence* property of grammatical words motivates a theory-based prediction regarding prearticulatory planning. Since grammatical words differ from lexical words in being dependent on a host, it can be hypothesized that the retrieval of a grammatical word has to be put on hold until its lexical host is available. The strongest version of this hypothesis is that the host expression has to be both selected and at least partially retrieved: intermediate versions are possible in which the nature of the lexical host can be less than fully specified when the grammatical element is produced. In either case, the choice of a grammatical element cannot be made independently of the choice of its lexical host, requiring a “detour” for grammatical elements. Partial processing of their lexical host is needed in order for grammatical words to be retrieved and encoded. Hence, it can be hypothesized that the planning of grammatical words is completed at a later stage than that of the lexical hosts of the grammatical words at hand.

This prediction is in line with psycholinguistic empirically-based models according to which grammatical encoding takes place at a later stage than lexical encoding [1, 2]. These models, which are largely based on the study of spontaneous production errors, distinguish three levels of language production: a semantic level, a sentence level and a motor level. The sentence level, which is the level of interest to this study, is further divided in a functional and a later positional stage. It is suggested that lexical words are retrieved at the functional stage, where they are specified as lemmas, while grammatical words are specified at the later positional stage. The positional stage is also the stage where the sentence frame is specified and where the previously retrieved lexical words will be placed.

In fact, the theory sketched above provides a theoretical motivation for such models: the dependence of grammatical words on hosts motivates that they are planned later than lexical words; lexical hosts presupposed by grammatical words are naturally planned before the grammatical words that presuppose them. This motivation is uni-directional: later planning is motivated by dependence, but dependence is not motivated by later planning.

In a wider perspective we suggest that the *dependence property* of grammatical words is a core reason why grammar is cognitively difficult to plan. This property prevents the speaker from planning one word at a time if a grammatical word is involved in the message. By virtue of the dependence property, grammatical words imply a retention mechanism which lexical words do not. Note that some lexical words are dependent, as in the case of adjectives that agree in gender and number with their head noun, but in this case the dependence relation is of a different kind as it is not a consequence of secondary (backgrounded) status of the adjective. It is thus a deviation from the purely incremental pathway that is in many other contexts the natural null hypothesis. Based on the dependence property, then, a *dependence hypothesis* for language production can be derived which covers two predictions. First, it is predicted that the planning of grammatical elements is associated with longer reaction times as compared to the planning of lexical elements. Second, it is predicted that the planning of grammatical items is associated with a lower accuracy rate. Note that the second of these predictions is shared with the prominence hypothesis (see above). The theory put forward in [4] does not only motivate the two psycholinguistic predictions, but also motivates a set of diagnostic criteria for distinguishing between lexical and grammatical items. Since only lexical items have the potential to express the main point of an utterance, only they can be singled out as the main point by means of focus markers or by being addressed in subsequent discourse. This entails that grammatical items are items that cannot individually be focalized or addressed [5]. In the experiment reported in this paper, all grammatical target items meet the criteria of non-focusability and non-addressability, and all lexical target items meet the opposite criteria of focusability and addressability.

### Methodological challenges

In addition to the theoretical challenges pointed out above, psycholinguistic studies contrasting grammatical and lexical words in language production face additional methodological ones.

#### Pinning down the contrast empirically

The first methodological challenge is that while grammatical words are the part of grammar that lends itself most easily to comparison with the lexicon, it is still not an easy task to contrast the two kinds of words. Lexical and grammatical words tend to have distinct distributions, and it is thus hard to find contexts in which the words can be contrasted. For instance, grammatical conjunctions cannot be replaced by lexical words, and most lexical verbs differ from auxiliaries in that only the latter require a co-occurring verb form. Lexical and grammatical words can of course be contrasted in isolation. This was done in a series of experiments initiated by [17], who found that open-class words were associated with shorter response times than closed-class words. Follow-up experiments however, did not replicate this finding [18, 19, 20, 21]. One-word production lacks an important property of grammatical words. Grammatical words do not exist in isolation, because, as discussed above, they are dependent on hosts [5]; see also [9] on the “lack of independent meaning” of English words such as *the* and *are*). In accordance with this, [22] found that words that do not occur in isolation are associated with longer response times than words that do, when contrasted in isolation. Later studies of item access embedded lexical decision tasks in sentences (presenting sentences one word at a time) and found either no difference between closed- and open-class words [23] or the opposite difference of Bradley’s [9].

#### Disentangling psycholinguistic factors

The second methodological challenge is that in English and other European languages that provide most of the psycholinguistic materials, the distinction under discussion here is bound up with other differences, including differences pertaining to word length, frequency and anticipation or predictability. For instance, grammatical words are most often more frequent and predictable than lexical ones. Such factors are known to affect naming latencies [24], which has been a clear confound in many studies. In an early study, [25] reported a frequency effect for lexical words which they fail to report for their grammatical counterparts. The authors used this finding to claim distinct routes for the two different item types. Later studies failed to reproduce this frequency effect, not only behavioural studies [19] but also several event related potential studies [26,27]. Similar issues have been reported for predictability. For instance, Segalowitz & Lane (2000) found that “no reading time advantage for closed-class words exists once Cloze value [reflecting anticipation] or log frequency [reflecting word frequency] are taken into account” [9]. Interestingly, durations of so-called function words and content words seem to be affected in a different way. In line with the prominence hypothesis we suggest, [28] reported shorter durations for function words compared to content words in conversational speech when frequency and predictability were controlled for. Additionally, content words presented shorter durations when more frequent while function words were not so affected. We will therefore examine the frequency and durations measures of grammatical vs. lexical words in the current study.

#### Determining the scope of planning

By insisting that the investigation of the contrast between grammatical words and lexical words should not involve words produced in isolation, we add a third challenge which is central to any study involving the production of more than one word: the question of planning, and of which incremental pattern drives the scope of encoding. To put it differently: when producing several words, how many words (or linguistic units) do speakers plan in advance and in which order are these encoded and retrieved before and during articulation?

As we will use reaction time measures of the onset of articulation, we need to make sure that the word of interest (bearing the contrast) has been encoded before articulation initiates in order to capture a potential effect. Unfortunately, the literature on how much speakers plan before they speak presents diverging results. Recent studies suggest that the span of encoding before articulation is not a fixed unit and that it can vary depending on different factors [29, 30, 31], the authors suggested that speakers strategically reduced their span of encoding in order to perform more accurately in a picture naming task where speakers produced simple adjective noun phrases. Interestingly, while the previous study focused on lexical words, different planning strategies have also been reported for grammatical words. For instance, [32] suggested that “function word repetition might stall speech so that the plan for a subsequent content word can be completed”. In so far as their “function words” overlap with grammatical words, this suggests that grammatical words also play a regulating role in speech planning and that when comparing contrasts with grammatical words and lexical words, different planning strategies are likely to be observed. Most importantly for the purpose of the current study, psycholinguistic models of speech production suggest that lexical elements are encoded and retrieved before grammatical elements [1]. If we assume that grammatical words are retrieved at a later stage than lexical ones and the span of encoding does not go beyond the first or two words of a message, RT measurements might not reflect a potential effect of a word being in the second position. If we do report a difference, however, this is strong evidence that in the current experiment, the span of encoding will comprise at least the first two words of the message. Additionally, we will analyse duration measures. RTs and articulation durations as complementary dependent variables were successfully used by [33] to investigate hierarchical planning in language production. Since duration measures might reflect planning mechanisms during articulation, this strategy should allow us to investigate part of the prominence hypothesis which predicts shorter durations and/or a lower accuracy rate for grammatical verbs.

#### Experimental strategy and design

In order to overcome the above mentioned challenges, we contrasted grammatical and lexical verbs which do not present such confounds. We contrasted grammatical vs. lexical verb pairs that are entirely homonymous, namely grammatical and lexical variants of the Danish verb forms *har* (‘has’) and *får* (‘gets’). While phonologically identical, grammatical and lexical instances of these verb forms can be distinguished based on their distributions (similarly to what is the case with variants of English *have*): the grammatical variants are auxiliaries that require a (possibly anaphorically represented) participle, just like English *has* in *Lise has missed a call*. In contrast, the lexical variants are transitive full verbs that require a (possibly anaphorically represented) NP object, as in the case of English *has* in *Lise has a missed call*.

Both grammatical and lexical instances of *har* and *får* can be repeated by means of the same verb form. This is different from English where lexical *have* can be repeated by means of do (*I have a dog*, *and so does she*). Repetition by means of the same verb form is the basis of our design. In order to keep predictability constant across conditions, the target words were elicited in a context of partial repetition where they appeared in exactly the same sentence frame and hence surrounded by the same words across conditions. Participants were first presented with sentences like those in (1) (with a grammatical target word) or (2) (with a lexical target word).

(1) *Lise har*gram *brugt en computer*. 'Lise has used a computer.'
(2) *Lise har*lex *en brugt computer*. 'Lise has a used computer.'

Each sentence formed a statement about a person and was followed by a question about a different person that the participant could reply to (3).

(3) *Hvad med Anne*? 'What about Anne?'

The replies elicited were of the type in (4) (grammatical) and (5) (lexical).

(4) *Det har*gram *hun også*. 'So has she'.
(5) *Det har*lex *hun også*. 'So does she'.

As mentioned, these replies constitute partial repetitions in which the target words appeared in exactly the same sentence frame and hence surrounded by the same words across conditions (cp. (4) and (5)). The only difference between the two conditions is found in the reference of *det* (corresponding to English *so*). *Det* is a Danish neuter pronoun, which in partially repetitive sentences like (4) and (5) represents neither an object (cf. the fact that *det* is neuter, while the object in (1) and (2), *en computer* 'a computer', is common gender) nor the complement part of a VP (cf. the fact that *det* is needed also in repetition of intransitive clauses: *Han løber*, *og det gør hun også* 'he is running, and so is she'), but a full VP (including any auxiliaries) (see Discussion section for more details).

If grammatical elements are dependent on the partial processing of their lexical host (in (4) represented by *det*) before they can be fully processed themselves (the dependence hypothesis), they should take longer time to plan. We should therefore observe longer reaction times for grammatical elements relative to lexical elements when comparing two perfectly matched sentences with only a difference in status (grammatical vs. lexical). Additionally, if grammatical words have low prominence compared to highly prominent lexical words (prominence hypothesis), they should present a lower processing cost (as reflected in their durations) once their processing can be fully carried out, or present a lower accuracy rate since they are less attended to. Even though the two hypotheses pull in opposite directions, we suggest that the dependence cost and prominence gain can be captured during different phases, respectively the planning (early stages) and the articulation (late stages) of the message with the additional information from the accuracy rate.

## Methods

### Materials

**Fig 1:**
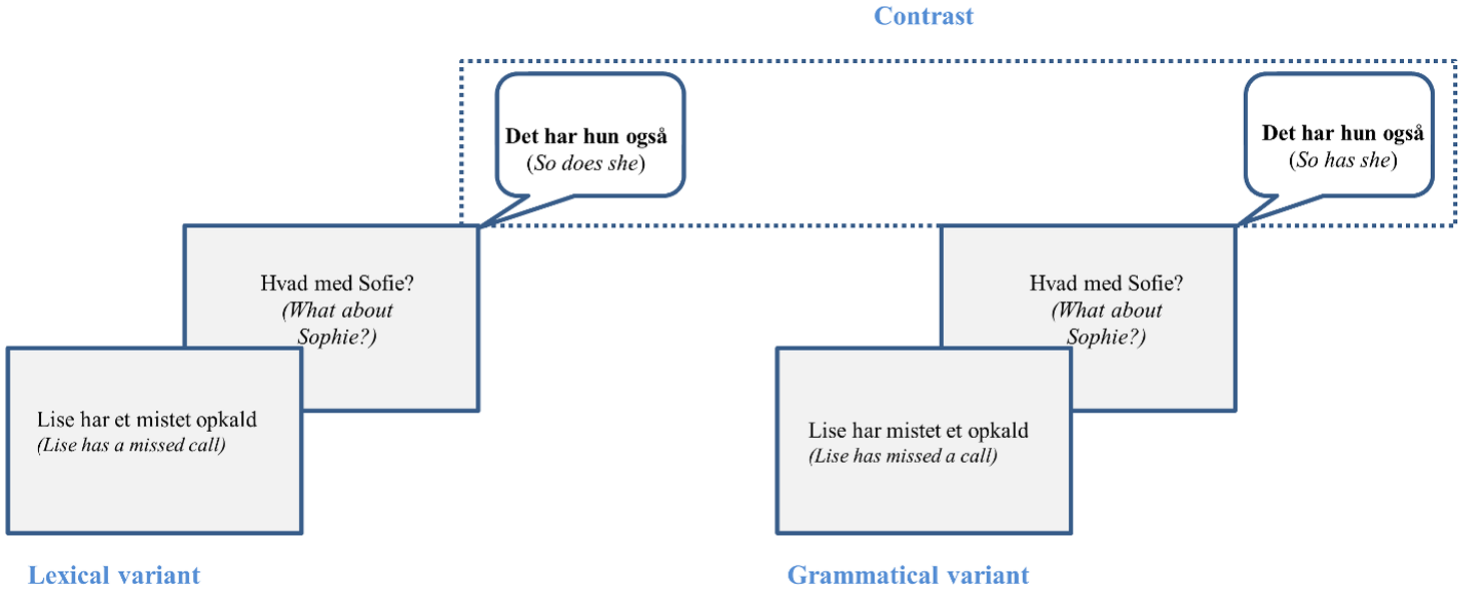
Trial example. Example of one trial in Experiment 1 in which the verb form *har* (‘has’) is elicited either as a lexical verb (full verb) or a grammatical verb (auxiliary), and the two verbs are compared in fully matched sentences (i.e. *Det har* lexical/grammatical *hun også* 'So does/haslexical/grammatical she').

The design used is illustrated in Fig 1 (see also description above). It was intended to approximate a natural communicative setting with question plus answer sequences.

Thirty sentences were created for each verb in each condition (lexical/grammatical). The list of sentences can be seen in S1 file. Ten male and ten female names were used in context sentences and questions in varying combinations, but always with the same gender for both context sentence and question. 120 filler sentences were constructed using two different neutral verbs which did not present a variant and added to the experiment. To ensure responses could not be planned in advance, complexity was increased by varying the polarity of the expected response. This was achieved by color-coding the question in blue or orange to elicit either a positive (6) or a negative (7) response. Participants saw both conditions of each item in either a positive or a negative frame. Four lists were created to balance which items were shown in a positive/negative frame, and which color was coded as positive/negative, across participants.

(6) *Det har*gram/lex *hun også*. 'So has/does she.'
(7) *Det har*gram/lex *hun ikke*. She has not/does not.'

**Fig 2:**
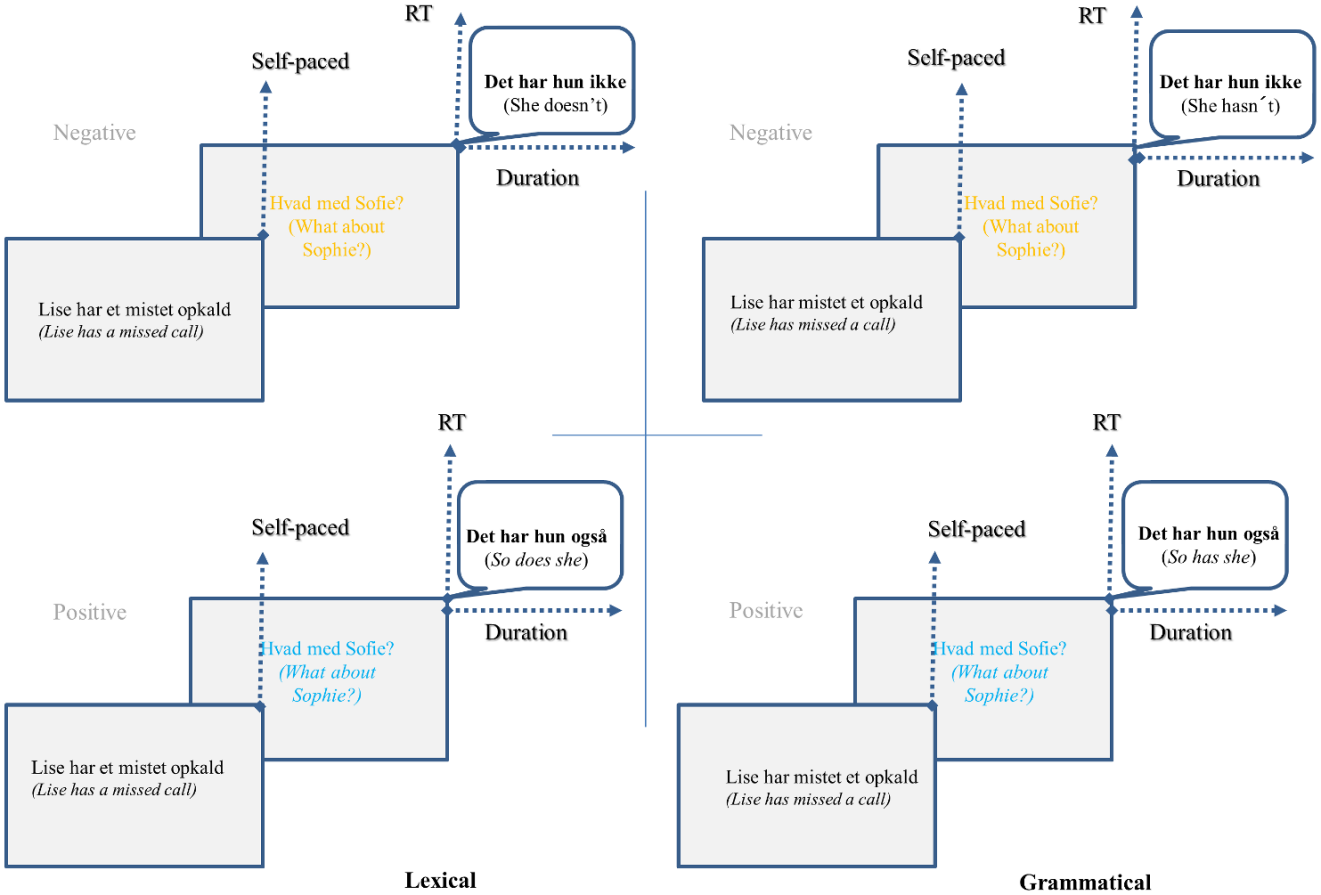
Design of the experiment. Each sequence starts with a context sentence which allows for the proper status (grammatical or lexical) to be elicited in the coming response. The color (blue or orange) indicates whether the participant should answer positively or negatively to the question. The dotted arrows represent the three types of measures used for the analyses with first self-paced measure upon reading the context sentence, then reaction time measures and eventually duration of the entire message.

Since the context sentences in the two conditions consisted of exactly the same words, the second condition presented to the participants may have been primed by the first. To control this, all lists were split in two, so half the items appeared on each sublist in one condition and on the other sublist in the other condition. The order of these sublists was balanced across participants as well. For each sublist the order of items was randomised for each participant. Each participant saw a total of 120 stimuli (60 grammatical and 60 lexical) and 120 filler trials. Filler trials consisted of sentences that contained different constructions with one of the two verbs: *blive* (‘become’) and *være* (‘be’). These verbs are comparable in term of frequency of use with *har* and *får* in Danish. Moreover, the auxiliary uses are clearly grammatical, while the copular uses are less clearly grammatical. The filler trials thus approximate the grammar-lexicon distinction found in the target trials. An overview of the overall design can be seen in Fig 2.

### Participants

Twenty four Danish speaking undergraduate students (12 females; mean age: 27 years) voluntarily took part in the experiment. One participant was removed from the analyses due to the repeated use of a verbum vicarium in one of the two conditions (e.g. use of the verb “gøre” instead of the verb “får”).

The study was approved by the Research Ethics committee at the Faculty of Humanities, University of Copenhagen.

### Procedure

The experiment was run in a quiet room on a laptop using Psychopy [34]. Prior to the experimental task, participants were given instructions and completed a practice session of four trials with the experimenter in the room to ensure the task had been understood. At the beginning of each trial, a context sentence was shown in the middle of the screen in black font on a grey background. The sentence remained on the screen until the participant clicked the spacebar. This was followed immediately by a question in either blue or orange font which was displayed for three seconds. Subjects were requested to provide either an affirmative or a negative response to the question based on the color of the font as quickly as possible. Participants wore a headset with a microphone to record the responses.

Recording of the participants response began at question onset and lasted five seconds after which a fixation cross appeared on the screen for one second before the next trial. The experiment took approximately 40 minutes to complete including a short break after the first 120 trials.

## Analyses

### 1. Estimate of the reading and comprehension time

In order to make sure that no possible reading differences between the grammatical context and the lexical context could account for a potential difference in the reaction times or duration, we measured the estimate of the reading and comprehension time by collecting self-paced measures of eliciting context sentences. Self-paced data was acquired by Psychopy software [34] by pressing the space bar. The condition (grammatical/lexical) was included in a linear mixed-effect model as fixed effect variable with participants and items as random effect variables. We calculated the error degree of freedom by subtracting the number of conditions from the number of observations. No difference was reported between the grammatical context (2215 ms; Standard deviation (SD) 962) and the lexical one (1990 ms; SD 974), p > 1. This result suggests that no reading differences in the context sentences might account for potential differences in reaction times or durations.

### 2. Polarity

Participants saw all trials for both conditions (grammatical and lexical) but each trial in one polarity only. To avoid potential polarity effects, lists were counterbalanced across participants. Even though the polarity was controlled for, we included it as a fixed effect variable in the final model using the Step function from the ‘lmerTest” package [35] in R. The Step function performs automatic backward elimination of all effects of linear mixed effect model. The purpose was to see whether adding polarity as a fixed effect improved the fit of the main model, but it did not. Since a potential polarity effect (or interaction with the polarity) was not part of our main hypothesis and did not improve the fit of the main model, we removed it from the final model presented below.

### 3. Verbs frequency

As no existing Danish database allows us to get frequency values for the two different forms of the verbs we use in this study, we extracted a sample of 500 occurrences from a large Danish reference corpus (KorpusDK1, Det Danske Sprog- og Litteraturselskab, 2007) and calculated the different occurrences for the grammatical and lexical form of each verbs. Values on Table 1 show that the grammatical variant for the verb “har” is more frequent than its lexical variant while the grammatical variant for the verb “får” is less frequent than its lexical counterpart, but no frequency effect is reported across conditions (p >1).

**Table 1:** Frequency of the different verb as grammatical and lexical occurrences calculated for a sample of 500 occurrences from the Danish reference corpus (KORPUSDK).

	Grammatical	Lexical	Unclassifiable	Total
**Har**	332	158	10	500
**Får**	54	372	74	500
**Average**	**193**	**265**	**42**	500*

***Note***: the total frequency of the occurrence of the verb *har* was of 552.604 out of 56 mio. and the total frequency for the verb *får* was of 59.810 out of 56 mio. words.

* http://ordnet.dk/korpusdk

### 4. Dependent variables

Reaction times were defined as the time elapsed between the onset of the visual presentation of the target question and the onset of the response (articulation). They were hypothesized to reveal a difference in planning before articulation due to the retention of the grammatical word. The measurement of naming latencies was operated by means of a voice key. In order to avoid any voice key failures when detecting the acoustic onset of the target word, all responses were systematically checked and corrected with speech analyser software (Praat [36]). Only correct responses were taken into account. As mixed models were used for the data analysis, we followed Baayen and Milin's [37] recommendation that only minimal data trimming be used. Only extreme outliers were discarded based on the visual distribution of the group, including correct trials only, thus preventing non-objective outlier removal. Reaction times above 2700 and below 200 ms for the remaining correct responses were removed from the dataset (2% and 3% for the grammatical and lexical condition respectively for the verb “har” and 2% and 2% for the verb “får”). Durations supposedly reflected encoding differences caused by prominence differences between the grammatical and lexical target word occurring during articulation. Durations were measured with speech analyser Software (Praat) from the acoustic onset of the first word until the offset of the last word of the message. Each sound file was manually segmented using visual cues from the oscillogram and auditive judgement. As for RTs, only correct responses were considered. Durations above 2500 ms and below 370 ms were discarded which corresponded to only three trials which all belonged to the grammatical condition and the verb “får”.

Accuracy rate were considered to be revealing of two phenomena. First, it supposedly reflects the attention attributed to each element with less attention being paid to grammatical/secondary elements (cf. the prominence hypothesis). Second, the fact that grammatical elements need to be put on hold during the partial processing of their lexical host, might also result to a lower accuracy rate for the production of grammatical versus lexical elements as it reflects a more complex process (cf. the dependence hypothesis). As errors were considered hesitations such as “uh”, “um” or a long silence anywhere in the message (1.81% for the grammatical (GRAM) and 1.67% for the lexical condition (LEX)), use of names instead of pronouns (1.88% for GRAM and 1.16% for LEX), use of the wrong polarity (2.03% for GRAM and 1.30% for LEX), use of a verbum vicarium instead of the target verb (1.74% for GRAM and 1.30% for LEX), use of the wrong verb (0.36% for GRAM and 0.22% for LEX), no responses (0.22% for GRAM and 0.43% for LEX), use of the wrong gender pronoun (0.07% for GRAM and 0.14% for LEX) and a single tense error in the grammatical condition. A total of 8.2% of errors was reported for GRAM and 6.2% for LEX. All error types were included in the analysis.

**Fig 3:**
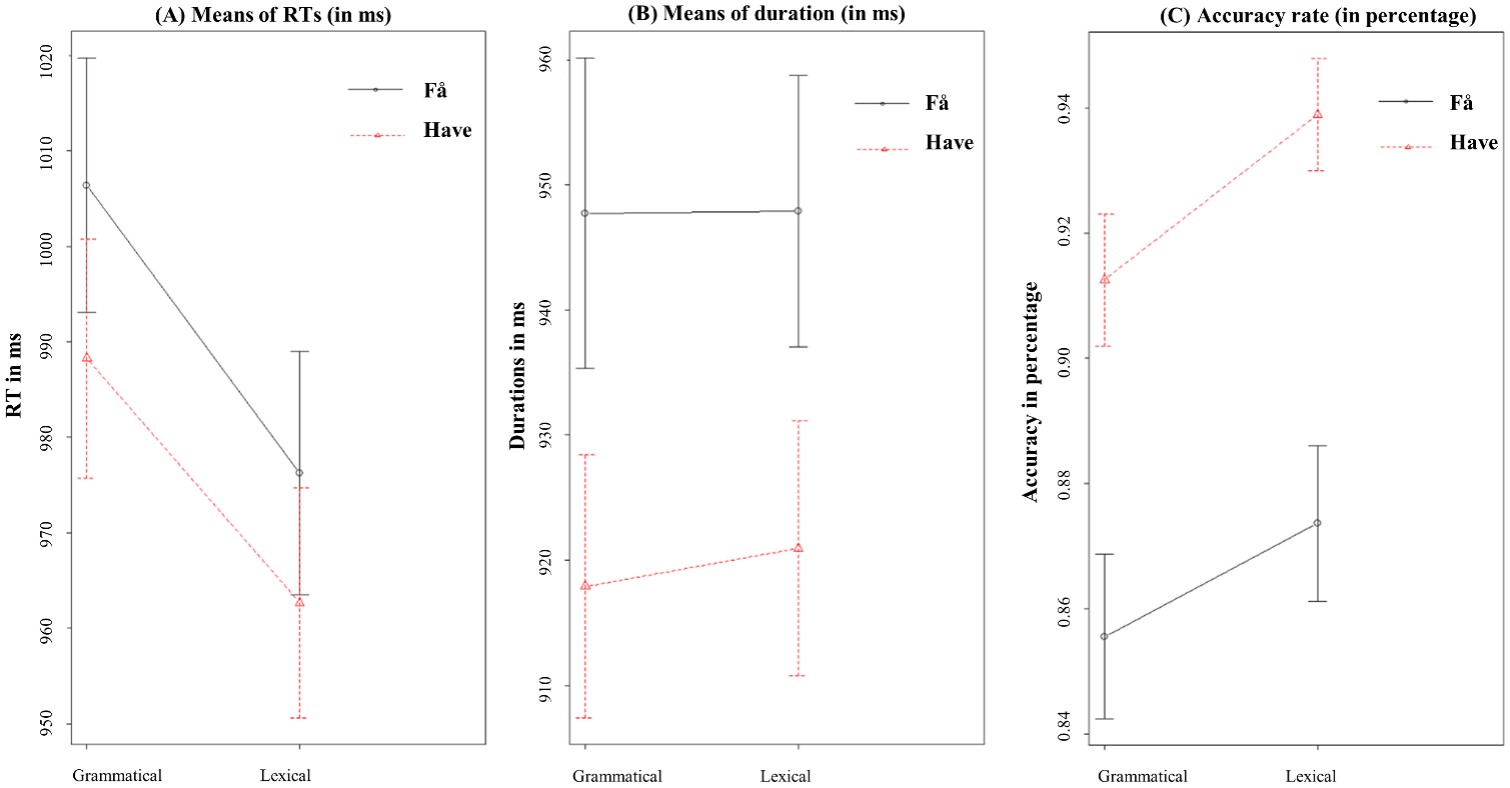
Means. Means of reactions times (A) in ms, durations (B) in ms, (and standard error of the mean in milliseconds) and accuracy in percentage (C) respectively for the verbs “får” and “har” for each condition (grammatical and lexical).

**Fig 4:**
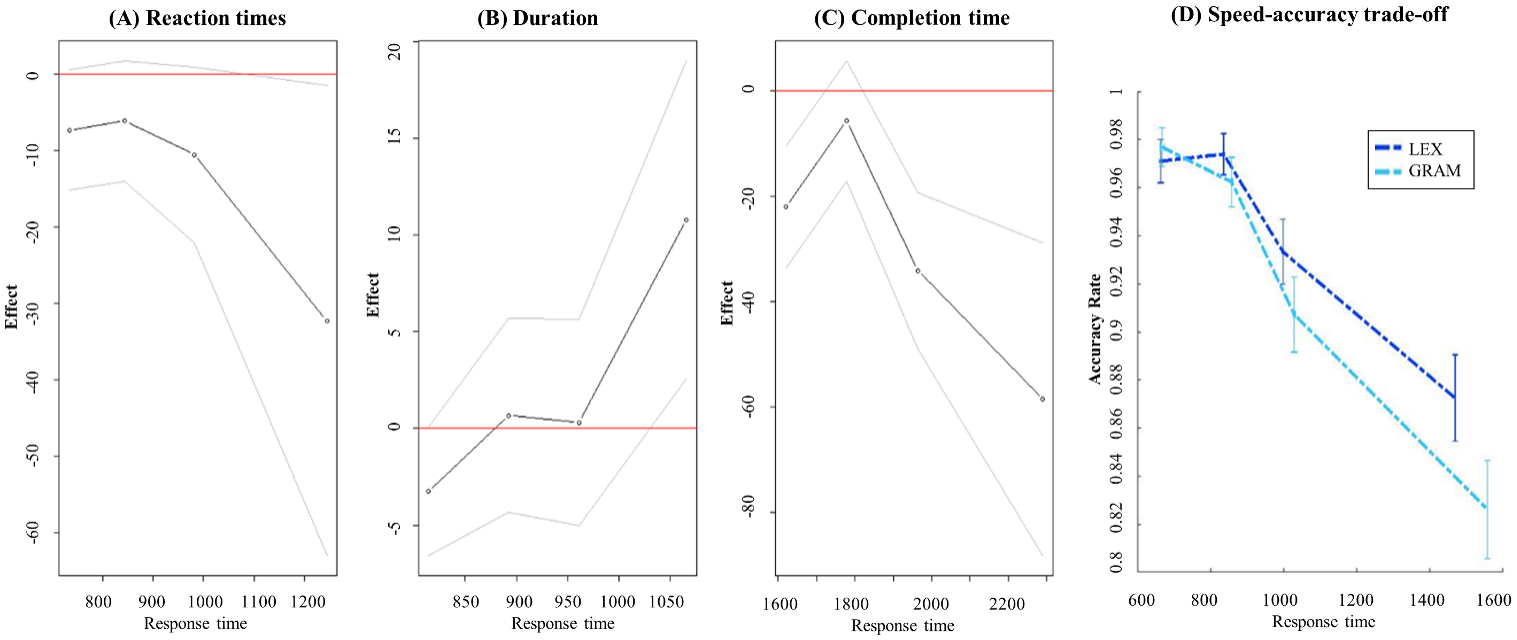
Delta plots A and B display the effect size (y-axis) corresponding to the subtraction of the means of the grammatical condition minus the means of the lexical condition. The effect size is plotted as a function of the response latencies divided in quartiles (x-axis). The red line indicates no difference, negative values indicate longer mean latencies for the grammatical condition and positive values indicate longer means for the lexical condition. Panel (C) represents the relation between speed and accuracy: the accuracy rate per condition (y-axis) is plotted against the response latencies divided in quartiles of the response latency distribution (x-axis).

An overview of all means for the three measures can be seen in Table 2 while results from the mixed models can be seen in Table 3. Additionally, plots of all means can be seen in Fig 3 and delta plot distributions of all variables can be seen in Fig 4.

**Table 2:** Overview of the means and SD in brackets for each type of measure (RT in ms, duration in ms and accuracy rate) for the main condition and each verb type (*får* and *har*).

RT	GRAM	LEX	Difference
Får	998 (310)	970 (258)	28
Har	990 (276)	967 (262)	23
Total	994 (345)	968 (318)	26
**Duration**			
Får	946 (121)	949 (118)	-3
Har	925 (122)	925 (115)	0
Total	935 (268)	937 (260)	-1
**Accuracy rate**			
Får	90%	92%	-2%
Har	93%	96%	-2%
Total	92%	94%	-2%

**Table 3:** Overview of the mixed model results for each type of measure (RT, duration and accuracy rate) for the main condition and the verb type (*får* and *har*).

**RT**	t value	p value
Condition	-2.25	0.026*
Verb type	-1.28	0.19
Condition*Verb type	0.71	0.47
**Duration**		
Condition	1.31	0.19
Verb type	-1.47	0.14
Condition*Verb type	-1.49	0.13
	z value	p value
**Accuracy rate**		
Condition	2.08	0.03*
Verb type	3.58	0.001*
Condition*Verb type	0.4	0.39

The * symbol in the p value column represents statistically significant values.

## Results

Mean latencies and durations were fitted with linear regression mixed models [37, 38] in two separate models (one for the RTs and one for the durations). All models included the main condition (grammatical/lexical) and the verb type (har/får) as fixed effect variables with participants and items as random effect variables. Accuracy rates were fitted with logit mixed-effects models [39] with the same random- and fixed-effects factors. The condition (grammatical/lexical) was included in the slopes of all three models but then removed as it did not improve the fitting level of the models.

As we predicted, reaction times revealed longer latencies for the sentences containing a grammatical verb (994 ms) relative to the sentences with a lexical verb (968 ms), t(2306) = -2.25, p < .026. We did not report an effect of the verb type nor an interaction of the verb type with the condition (ps>1). The accuracy rate was lower in the grammatical condition (91.8%) relative to the lexical one (93.8%), z(2759) = 2.08, p< 0.03. We also report a lower accuracy rate for the verb “får” (91%) relative to the verb “har” (94%) but no interaction with the condition (p>1). Contrary to our hypothesis, durations did not present any difference across conditions (t<1). The durations of the verbs only was also measured and analysed as a posthoc analysis but no difference was found across conditions.

At this stage of the analysis, it is not possible to disentangle whether the difference in accuracy rate is reflecting the dependence property or the prominence property of grammatical words since both properties might have led to a lower accuracy rate for the grammatical condition. In other words, since both the prominence hypothesis and the dependence hypothesis predict lower production accuracy for grammatical elements as compared to lexical ones, our findings with respect to accuracy support both hypotheses. In order to disentangle the two possible accounts, we exploited the fact that duration differences are predicted only by the prominence hypothesis, while RT differences are predicted only by the dependence hypothesis and compared the distribution of the accuracy rate with the distribution of the RT and duration respectively. We therefore examined effect sizes as a function of response times by means of delta plot distributions for RT and duration. A similarity between accuracy and duration patterns would suggest that, just like duration differences, accuracy differences should be accounted for in terms of the prominence property of grammatical items. In contrast, a similarity between accuracy and RT patterns would suggest that, just like RT differences, accuracy differences should be accounted for in terms of the dependence property of grammatical items.

The delta plot analyses rest on expectations that speakers present variation when planning speech, as a result of which the effect of grammatical vs. lexical status may be modulated by, for instance, response time (which supposedly reflects planning strategies). Thus, we plotted the effect of each main variable as a function of the response times divided into quartiles. Fig 4A represents the effect of the main condition (grammatical/lexical) as a function of the reaction times; Fig 4B, the main effect as a function of the durations; and Fig 4C the accuracy rate for each condition as a function of the response times (note that 9 trials which corresponded to no answer were removed from Fig 4C as they did not have a corresponding RT). As can be observed in all four figures, the effect predicted by the theory seems to be best represented by longer response times. Remarkably, the duration effect which we failed to observe (shorter latencies for the grammatical responses) seems to follow the predicted pattern but only for the longest responses. The same observation is also striking for the accuracy rate. Most interestingly, the accuracy rate seems to follow precisely the same pattern as the reaction time distribution. This suggests that, just like the RT difference between the grammatical and lexical conditions, the accuracy difference seems to be reflecting predictions made by the dependency property of grammatical elements. Based on the observation that the overall effects seem to be stronger in general for longer response times, we decided to split the data into short responses and long responses as based on the overall speed of the participants giving a short group with an average of 846 ms and a long group with an average of 1119 ms. This left one participant out (mean = 1004ms) as we needed an equal number in each group. We then examined potential interactions between main effect and speed for all four variables. None of the interactions reached significance (all t*s*<1).

## Discussion

The purpose of this study was to investigate whether grammatical verbs and lexical verbs are planned and produced differently when otherwise fully controlled for potential psycholinguistic confounds. The contrasts elicited were minimal: i) grammatical and lexical words were homonymous ii) grammatical and lexical verbs were contrasted in identical sentences. Importantly, grammatical and lexical verbs were contrasted in multi-word sentences in a conversation-like setup. Where most models of speech production are based on single words, this setup allowed to mirror spontaneous speech production. Importantly, the selection of the grammatical and lexical words that were tested was based on theoretically motivated criteria [5], as opposed to theoretically unanchored distinctions between “closed” and “open-class words” or “function” and “content words”. Based on the theory in [5], we tested two predictions:

### Low prominence prediction

The first one was called the *low prominence prediction*. We predicted that the articulation of grammatical words is shorter and/or less accurate than the articulation of lexical words, as grammatical words, being secondary, are less crucial for communicative purposes. We do report a lower accuracy rate for grammatical elements which, we hypothesized, might have reflected the low prominence hypothesis. Nevertheless, the fact that the distribution of the accuracy rate as a function of response time (Fig 4C) mirrors exactly the distribution of the RT as a function of response time (Fig 4A), suggests that the lower accuracy rate actually relates to the dependence feature more than to the prominence one. Interestingly, while speed-accuracy trade-off usually predicts a lower accuracy rate for fast responses, our results indicate that the longer speakers plan before articulation, the more errors they are likely to produce. A simple explanation for this observation is based on the assumption that longer response times reflect more pre-articulatory planning: the longer the response time, the more words planned prior to articulation and therefore the more room for errors.

The low prominence prediction was not reflected in the durations as expected. Even though the delta distribution of the duration measure (Fig 4B) suggests a tendency for durations to decrease for grammatical elements specifically for the longer response times, we could not provide statistical support for this claim. Failure to observe differences in durations might come from the fact that the same verbs were repeated all over again with too little variation in the response. Contrary to us, [28] did report shorter durations for function words compared to content words in their study where they examined conversational speech with enough variation in the material to capture a potential duration difference. All in all, we did not verify the prominence hypothesis as no difference in duration was reported and the distribution of the accuracy rate as a function of the reaction times highly suggests that the accuracy rate relates to the dependence feature.

### Dependence prediction

The dependence prediction was derived both from the linguistic theory by Boye and Harder (2012) and from speech production models [1, 2]. As described in the introduction, existing sentence production models claim that grammatical and lexical words are processed at different levels and stages with the retrieval of lexical words at the functional stage followed by the specification of grammatical words and insertion of lexical ones at the positional stage. Similarly, the linguistic theory by [5], predicts that the planning of grammatical words is completed at a later stage than that of the lexical hosts of the grammatical words at hand, as partial processing of their lexical host is needed in order for grammatical word to be retrieved and encoded. Thus, while lexical words can be processed in a rather straightforward fashion, grammatical words require that other (lexical) words be encoded, retrieved and inserted before they (grammatical words) can be specified. In our study, we compared well matched sentences with a difference in verb status, one condition involving grammatical verbs and the other lexical verbs. Based on the above-mentioned models and theory, we hypothesized that the sentences containing a grammatical verb require a more complex processing than the sentences containing a lexical one. A more complex processing can translate into either longer naming latencies, a higher error rate or both.

In line with the dependence hypothesis, we reported longer naming latencies for the planning of grammatical verbs relative to lexical verbs. Interestingly, this observation cannot be accounted for as reflecting frequency differences as both verb types investigated show longer RTs for the grammatical condition despite having very different frequency distributions: in the case of *har*, the grammatical variant is more frequent than the lexical one, but in the case of *få*, the grammatical variant is less frequent than the lexical one. This suggests that the condition effect observed is driven mostly by processing differences and not frequency differences. These results are in line with earlier studies which did not find a difference in frequency effect between grammatical and lexical words [19, 26, 27]. Moreover, the two verb types investigated showed different accuracy patterns with a lower accuracy rate for the verb type *får* (91%) relative to the verb type *har* (94%), which did not affect the main effect. Thus, the production of grammatical verb variants presents longer processing time than the production of lexical verb variants in spite of the fact that the two verb types investigated, *har* and *får*, differ both in terms of frequency patterns and difficulty of use. As reported above, our findings with respect to accuracy rate also seem to be in line with the dependence hypothesis.

### PAst DIscourse LInking Hypothesis

It is important to note that also a different account would predict longer reaction times for auxiliaries compared to lexical verbs. The PAst DIscourse LInking Hypothesis (PADILIH; [40]) would indeed predict a cost of encoding for verbs that relate to a past event. The grammatical auxiliary *har* here relates to a past event: as a perfect auxiliary, it relates to an anterior event of relevance to the speech situation. In contrast, lexical *har* does not relate to a past event but simply to a present event. Thus, in accordance with the PADILIH, the longer RTs for grammatical *har* relative to lexical *har* might be due to this difference. However, this alternative explanation is not available in the case of *får*, the other verb studied in the experiment, because the grammatical variant of *får* does not relate to a past event. There is no obvious difference between the lexical and the grammatical variants of *får* in terms of temporal reference potential (which can be either present or future). Therefore, longer reaction times for the grammatical variant of *får* can be explained by the dependence hypothesis developed in this paper, but not by a link to a past event. Taken together, our results support the assumption that the property of being a grammatical word is more determining than the word type as such and that PADILIH alone cannot account for our results.

### A second difference between the grammatical and the lexical condition

With predictability differences controlled for in the experimental setting, and with frequency and time-reference differences effectively ruled out (see above), we see only one possible alternative way of accounting for the RT and accuracy differences observed between the grammatical and lexical condition. As described earlier, matching of the two conditions was based on a context of partial repetition in which pronouns anaphorically represent the VPs that distinguish grammatical verb variants from lexical ones. Participants first read sentences like those in (1) (grammatical condition) or (2) (lexical condition), then a question like the one in (3), to which they answered using sentences like those in (4) (grammatical condition) or (5) (lexical condition) (examples are repeated here for convenience).

(1) *Lise har*gram *brugt en computer*. 'Lise has used a computer.'
(2) *Lise har*lex *en brugt computer*. 'Lise has a used computer.'
(3) *Hvad med Anne*? 'What about Anne?'
(4) *Det har*gram *hun også*. 'So has she'.
(5) *Det har*lex *hun også*. 'So does she'.

In (4), the pronoun (*det*) represents the VP (including any auxiliaries) in (1): *har*gram *brugt en computer* ('has used a computer'); in (5), it represents the VP in (2): *har*lex *en brugt computer* ('has a used computer'). The VPs in (4) and (5) contain the same number of words and morphemes, but there is a difference between them: the VP in (1), represented by *det* in (4), consists of an auxiliary, a full verb and an object NP consisting of an indefinite article and a noun; the VP in (2) represented by *det* in (5) consists of a full verb and an object NP consisting of an indefinite article, a noun and an attributive adjective. This represents an (in the context of verb-based experiments) unavoidable possible confound. The difference between the anaphorically represented VPs might trigger the RT and accuracy differences observed. More specifically, it cannot be ruled out that it is more difficult (as reflected in RT and accuracy differences) to anaphorically represent the former VP than the latter. There is no straightforward theoretical rationale for assuming this, however. Moreover, the difference boils down to a difference which closely mirrors the difference between grammatical and lexical elements that our study was designed to examine: In the grammatical condition, the "extra" element in the VP is a grammatical element, i.e. the auxiliary *har*gram; in the lexical condition, the "extra" element in the VP is a lexical element, i.e. an attributive adjective such as *brugt* ('used') in (2).

### Limitations

The nature or locus of the RT differences is difficult to pin down. Differences in RTs can be the result of a longer process or of an additional process. The current study does not allow us to provide more information on whether the longer RTs for the grammatical condition are the results of an additional process or a longer process. We would like to claim that grammatical words take longer to process because of a retention phenomenon (where the lexical host has to be at least partially processed for the grammatical word to be retrieved and processed) but a longer reaction time might simply reflect a different processing strategy. For instance, grammatical and lexical words might involve exactly the same processing route, differing only in terms of timing, grammatical words needing more time than lexical ones due to their secondary status. Differences in terms of representation but not processing route might indeed also lead to RT differences. This study cannot provide support for one specific scenario.

## Conclusion

Taken together, the results of this study clearly demonstrate differences between the planning of grammatical and lexical verbs during speech production in an optimally designed contrast. Longer RTs for the grammatical condition as well as a lower accuracy rate suggest a later or more difficult planning for grammatical verbs vs. lexical verbs in line with sentence production models. Interestingly, frequency differences did not influence naming latencies which indicates that the status of the verb (grammatical or lexical) plays a more significant role than frequency in the planning of multi-word sentences. Further work on similar contrasts but different word types is needed in order to shed light on the grammar/lexicon distinction not simply at the word level but also rule level.

## Supporting information

S1 Fig Material. List of the thirty sentences created for each verb in each condition (lexical/grammatical).
(DOCX)
