# Supplementary material for: Planning and production of grammatical and lexical verbs in multi-word messages
Source: PLoS One. 2017 Nov 1;12(11):e0186685. doi: 10.1371/journal.pone.0186685 (PMC5665509; doi:10.1371/journal.pone.0186685)
Supplement: S1 Fig — List of the thirty sentences created for each verb in each condition (lexical/grammatical). (DOCX) [file pone.0186685.s001.docx]

### Target stimuli Experiment 1

| **Lexical condition** | **Grammatical condition** |
| --- | --- |
| *Lise har et mistet opkald.*  'Lise has a missed call.' | *Lise har mistet et opkald.*  'Lise has missed a call.' |
| *Hanne har en arvet spillegæld.*  'Hanne has an inherited gambling debt.' | *Hanne har arvet en spillegæld.*  'Hanne has inherited a gambling debt.' |
| *Pia har en beundret kunstsamling.*  'Pia has an admired art collection.' | *Pia har beundret en kunstsamling.*  'Pia has admired an art collection.' |
| *Maja har en brugt computer.*  'Maja has a used computer.' | *Maja har brugt en computer.*  'Maja has used a computer.' |
| *Tina har en designet brudekjole.*  'Tina has a designed wedding dress.' | *Tina har designet en brudekjole.*  'Tina has designed a wedding dress.' |
| *Jesper har en fortjent placering.*  'Jesper has a deserved position.' | *Jesper har fortjent en placering.*  'Jesper has deserved a position.' |
| *Anders har en knækket fortand.*  'Anders has a cracked front tooth.' | *Anders har knækket en fortand.*  'Anders has cracked a front tooth.' |
| *Ole har et kogt æg.*  'Ole has a boiled egg.' | *Ole har kogt et æg.*  'Ole has boiled an egg.' |
| *Thomas har et ladt gevær.*  'Thomas has a loaded rifle.' | *Thomas har ladt et gevær.*  'Thomas has loaded a rifle.' |
| *Søren har en lydisoleret væg.*  'Søren has a soundproofed wall.' | *Søren har lydisoleret en væg.*  'Søren has soundproofed a wall.' |
| *Bente har en ødelagt harddisk.*  'Bente has a broken hard disk.' | *Bente har ødelagt en harddisk.*  'Bente has broken a hard disk.' |
| *Karen har en opvarmet pizza.*  'Karen has a heated pizza.' | *Karen har opvarmet en pizza.*  'Karen has heated a pizza.' |
| *Lone har en pumpet bold.*  'Lone has an inflated ball.' | *Lone har pumpet en bold.*  'Lone has inflated a ball.' |
| *Marie har en røget laks.*  'Marie has a smoked salmon.' | *Marie har røget en laks.*  'Marie has smoked a salmon.' |
| *Mette har en skjult skat.*  'Mette has a hidden treasure.' | *Mette har skjult en skat.*  'Mette has hidden a treasure.' |
| *Henrik har en smadret lampe.*  'Henrik has a shattered lamp.' | *Henrik har smadret en lampe.*  'Henrik has shattered a lamp.' |
| *Martin har en stjålet cykel.*  'Martin has a stolen bike.' | *Martin har stjålet en cykel.*  'Martin has stolen a bike.' |
| *Morten har en trænet politihund.*  'Morten has a trained police dog.' | *Morten har trænet en politihund.*  'Morten has trained a police dog.' |
| *Peter har en udstoppet ræv.*  'Peter has a stuffed fox.' | *Peter har udstoppet en ræv.*  'Peter has stuffed a fox.' |
| *Emil har en udviklet sans for mode.*  'Emil has a developed sense of fashion.' | *Emil har udviklet en sans for mode.*  'Emil has developed a sense of fashion.' |
| *Lise har en reserveret plads.*  'Lise has a reserved seat.' | *Lise har reserveret en plads.*  'Lise has reserved a seat.' |
| *Hanne har en planlagt fridag.*  'Hanne has a planned day off.' | *Hanne har planlagt en fridag.*  'Hanne has planned a day off.' |
| *Pia har en lejet lejlighed.*  'Pia has a rented apartment.' | *Pia har lejet en lejlighed.*  'Pia has rented an apartment.' |
| *Maja har en underskrevet kontrakt.*  'Maja has a signed contract.' | *Maja har underskrevet en kontrakt.*  'Maja has signed a contract.' |
| *Tina har et malet portræt af Dronningen.*  'Tina has a painted portrait of the Queen.' | *Tina har malet et portræt af Dronningen.*  'Tina has painted a portrait of the Queen.' |
| *Jesper har en ændret udgave af programmet.*  'Jesper has a changed version of the program.' | *Jesper har ændret en udgave af programmet.*  'Jesper has changed a version of the program.' |
| *Anders har en syltet agurk.*  'Anders has a pickled cucumber.' | *Anders har syltet en agurk.*  'Anders has pickled a cucumber.' |
| *Ole har et beskadiget sidespejl.*  'Ole has a damaged a side view mirror.' | *Ole har beskadiget et sidespejl.*  'Ole has damaged a side view mirror.' |
| *Thomas har en asfalteret indkørsel.*  'Thomas has an asphalted driveway.' | *Thomas har asfalteret en indkørsel.*  'Thomas has aphalted a driveway.' |
| *Søren har en trådet nål.*  'Søren has a threaded needle.' | *Søren har trådet en nål.*  'Søren has threaded a needle.' |
| *Bente får en blandet pose slik.*  'Bente gets a mixed bag of candy.' | *Bente får blandet en pose slik.*  'Bente gets a bag of mixed candy.' |
| *Karen får en farvet t-shirt.*  'Karen gets a coloured t-shirt.' | *Karen får farvet en t-shirt.*  'Karen gets a t-shirt coloured.' |
| *Lone får en foldet servietsvane.*  'Lone gets a folded napkin swan.' | *Lone får foldet en servietsvane.*  'Lone gets a napkin swan folded.' |
| *Marie får et forbedret styresystem.*  'Marie gets an improved operating system.' | *Marie får forbedret et styresystem.*  'Marie gets an operating system improved.' |
| *Mette får en forgyldt ring.*  'Mette gets a gold plated ring.' | *Mette får forgyldt en ring.*  'Mette gets a ring gold plated.' |
| *Henrik får en frankeret svarkuvert.*  'Henrik gets a stamped addressed envelope.' | *Henrik får frankeret en svarkuvert.*  'Henrik gets an addressed envelope stamped.' |
| *Martin får en fyldt kande med saft.*  'Martin gets a filled pitcher with juice.' | *Martin får fyldt en kande med saft.*  'Martin gets a pitcher with juice filled.' |
| *Morten får et garvet skind.*  'Morten gets a tanned hide.' | *Morten får garvet et skind.*  'Morten gets a hide tanned.' |
| *Peter får en gødet plante.*  'Peter gets a fertilized plant.' | *Peter får gødet en plante.*  'Peter gets a plant fertilized.' |
| *Emil får en godkendt ansøgning.*  'Emil gets an approved application.' | *Emil får godkendt en ansøgning.*  'Emil gets an application approved.' |
| *Lise får en halveret melon.*  'Lise gets a halved melon.' | *Lise får halveret en melon.*  'Lise gets a melon halved.' |
| *Hanne får et indrammet foto.*  'Hanne gets a framed photo.' | *Hanne får indrammet et foto.*  'Hanne gets a photo framed.' |
| *Pia får en kodet meddelelse.*  'Pia gets an encoded message.' | *Pia får kodet en meddelelse.*  'Pia gets a message encoded.' |
| *Maja får en krydret ret.*  'Maja gets a seasoned dish.' | *Maja får krydret en ret.*  'Maja gets a dish seasoned.' |
| *Tina får et lamineret diplom.*  'Tina gets a laminated certificate.' | *Tina får lamineret et diplom.*  'Tina gets a certificate laminated.' |
| *Jesper får et nedskrevet manifest.*  'Jesper gets a written manifesto.' | *Jesper får nedskrevet et manifest.*  'Jesper gets a manifesto written.' |
| *Anders får en hæklet grydelap.*  'Anders gets a crocheted potholder.' | *Anders får hæklet en grydelap.*  'Anders gets a potholder crocheted.' |
| *Ole får en dekoreret æske.*  'Ole gets a decorated box.' | *Ole får dekoreret en æske.*  'Ole gets a box decorated.' |
| *Thomas får en saltet skinke.*  'Thomas gets a salted ham.' | *Thomas får saltet en skinke.*  'Thomas gets a ham salted.' |
| *Søren får en samlet LEGO-borg.*  'Søren gets an assembled LEGO castle.' | *Søren får samlet en LEGO-borg.*  'Søren gets a LEGO castle assembled.' |
| *Bente får en låst telefon.*  'Bente gets a locked phone.' | *Bente får låst en telefon.*  'Bente gets a phone locked.' |
| *Karen får et strikket halstørklæde.*  'Karen gets a knitted scarf.' | *Karen får strikket et halstørklæde.*  'Karen gets a scarf knitted.' |
| *Lone får en strøget skjorte.*  'Lone gets an ironed shirt.' | *Lone får strøget en skjorte.*  'Lone gets a shirt ironed.' |
| *Marie får en trykt sangtekst.*  'Marie gets a printed set of lyrics.' | *Marie får trykt en sangtekst.*  'Marie gets a set of lyrics printed.' |
| *Mette får en marineret sild.*  'Mette gets a marinated herring.' | *Mette får marineret en sild.*  'Mette gets a herring marinated.' |
| *Henrik får en stegt and.*  'Henrik gets a fried duck.' | *Henrik får stegt en and.*  'Henrik gets a duck fried.' |
| *Martin får en kopieret cd.*  'Martin gets a copied cd.' | *Martin får kopieret en cd.*  'Martin gets a cd copied.' |
| *Morten får et eftersendt brev.*  'Morten gets a forwarded letter.' | *Morten får eftersendt et brev.*  'Morten gets a letter forwarded.' |
| *Peter får en lakeret kommode.*  'Peter gets a lacquered dresser.' | *Peter får lakeret en kommode.*  'Peter gets a dresser lacquered.' |
| *Emil får en betalt rejse.*  'Emil gets a paid trip.' | *Emil får betalt en rejse.*  'Emil gets a trip paid.' |

### Filler stimuli Experiment 1

| **Construction type 1** | **Construction type 2** |
| --- | --- |
| *Lise er en tilbedt stjerne.*  'Lise is a worshipped star.' | *Lise er tilbedt som stjerne.*  'Lise is worshipped as a star.' |
| *Hanne er en hyret musiker.*  'Hanne is a hired musician.' | *Hanne er hyret som musiker.*  'Hanne is hired as a musician.' |
| *Pia er en udpeget diplomat.*  'Pia is an appointed diplomat.' | *Pia er udpeget som diplomat.*  'Pia is appointed as a diplomat.' |
| *Maja er en valgt repræsentant.*  'Maja is a chosen representative.' | *Maja er valgt som repræsentant.*  'Maja is chosen as a representative.' |
| *Tina er en forklædt nazist.*  'Tina is a disguised nazi.' | *Tina er forklædt som nazist.*  'Tina is disguised as a nazi.' |
| *Jesper er en uddannet lærer.*  'Jesper is an educated teacher.' | *Jesper er uddannet som lærer.*  'Jesper is educated as a teacher.' |
| *Anders er en udlært tømrer.*  'Anders is a trained carpenter.' | *Anders er udlært som tømrer.*  'Anders is trained as a carpenter.' |
| *Ole er en sminket klovn.*  'Ole is a madeup clown.' | *Ole er sminket som klovn.*  'Ole is madeup as a clown.' |
| *Thomas er en trænet højdespringer.*  'Thomas is a practised high jumper.' | *Thomas er trænet som højdespringer.*  'Thomas is practised as a high jumper.' |
| *Søren er en udstationeret militærlæge.*  'Søren is a stationed military doctor.' | *Søren er udstationeret som militærlæge.*  'Søren is stationed as a military doctor.' |
| *Bente er en overset digter.*  'Bente is an overlooked poet.' | *Bente er overset som digter.*  'Bente is overlooked as a poet.' |
| *Karen er en undervurderet arbejdskraft.*  'Karen is an underappreciated worker.' | *Karen er undervurderet som arbejdskraft.*  'Karen is underappreciated as a worker.' |
| *Lone er en anerkendt skuespiller.*  'Lone is an acknowledged actor.' | *Lone er anerkendt som skuespiller.*  'Lone is acknowledged as an actor.' |
| *Marie er en afholdt dronning.*  'Marie is a cherised queen.' | *Marie er afholdt som dronning.*  'Marie is cherised as a queen.' |
| *Mette er et hædret æresmedlem.*  'Mette is a celebrated honorary member.' | *Mette er hædret som æresmedlem.*  'Mette is celebrated as an honorary member.' |
| *Henrik er en udnævnt æresdoktor.*  'Henrik is an appointed honorary doctor.' | *Henrik er udnævnt som æresdoktor.*  'Henrik is appointed as honorary doctor.' |
| *Martin er en konstitueret leder.*  'Martin is an acting manager.' | *Martin er konstitueret som leder.*  'Martin is acting as a manager.' |
| *Morten er en accepteret ekspert.*  'Morten is an accepted expert.' | *Morten er accepteret som ekspert.*  'Morten is accepted as an expert.' |
| *Peter er en hædret vinder.*  'Peter is an honored winner.' | *Peter er hædret som vinder.*  'Peter is honered as a winner.' |
| *Emil er en maskeret kat.*  'Emil is a masked cat.' | *Emil er maskeret som kat.*  'Emil is masked as a cat.' |
| *Lise er en registreret forbryder.*  'Lise is a registered criminal.' | *Lise er registreret som forbryder.*  'Lise is registered as a criminal.' |
| *Hanne er en fængslet morder.*  'Hanne is an imprisoned murderer.' | *Hanne er fængslet som morder.*  'Hanne is imprisoned as a murderer.' |
| *Pia er en egnet donor.*  'Pia is a well-suited donor' | *Pia er egnet som donor.*  'Pia is well-suited as a donor.' |
| *Maja er en afsløret svindler.*  'Maja is an exposed hustler.' | *Maja er afsløret som svindler.*  'Maja is exposed as a hustler.' |
| *Tina er en beskæftiget håndværker.*  'Tina is an employed craftsperson.' | *Tina er beskæftiget som håndværker.*  'Tina is employed as a craftsperson.' |
| *Jesper er en svækket formand.*  'Jesper is a weakened chairman.' | *Jesper er svækket som formand.*  'Jesper is weakened as a chairman.' |
| *Anders er en værdsat dommer.*  'Anders is an appreciated judge.' | *Anders er værdsat som dommer.*  'Anders is appreciated as a judge.' |
| *Ole er en anklaget morder.*  'Ole is an accused murderer.' | *Ole er anklaget som morder.*  'Ole is accused as a murderer.' |
| *Thomas er en fejret forfatter.*  'Thomas is a celebrated author.' | *Thomas er fejret som forfatter.*  'Thomas is celebrated as an author.' |
| *Søren er en ønsket samarbejdspartner.*  'Søren is a wanted collaborator.' | *Søren er ønsket som samarbejdspartner.*  'Søren is wanted as a collaborator.' |
| *Bente bliver i et afkølet bassin.*  'Bente remains in a cooled pool.' | *Bente bliver afkølet i et bassin.*  'Bente becomes/is cooled in a pool.' |
| *Karen bliver i et afslappet forhold.*  'Karen remains in a relaxed relationship.' | *Karen bliver afslappet i et forhold.*  'Karen becomes/is relaxed in a relationship.' |
| *Lone bliver i et anerkendt politisk parti.*  'Lone remains in a recognized political party.' | *Lone bliver anerkendt i et politisk parti.*  'Lone becomes/is recognized in a political party.' |
| *Marie bliver hos en autoriseret revisor.*  'Marie remains with a certified accountant.' | *Marie bliver autoriseret hos en revisor.*  'Marie becomes/is certified with an accountant.' |
| *Mette bliver i en begejstret teatertrup.*  'Mette remains in an excited theater company.' | *Mette bliver begejstret i en teatertrup.*  'Mette becomes/is excited in a theater company.' |
| *Henrik bliver i en beskyttet institution.*  'Henrik remains in a protected facility.' | *Henrik bliver beskyttet i en institution.*  'Henrik becomes/is protected in a facility.' |
| *Martin bliver hos en beundret designer.*  'Martin remains with an admired designer.' | *Martin bliver beundret hos en designer.*  'Martin becomes/is admired with a designer.' |
| *Morten bliver hos en efterspurgt modefotograf.*  'Morten remains with a sought-after fashion photographer.' | *Morten bliver efterspurgt hos en modefotograf.*  'Morten becomes/is sought-after with a photographer.' |
| *Peter bliver i et engageret bofællesskab.*  'Peter remains in a dedicated flatshare.' | *Peter bliver engageret i et bofællesskab.*  'Peter becomes/is dedicated in a flatshare.' |
| *Emil bliver i en interesseret gruppe.*  'Emil remains in an interested group.' | *Emil bliver interesseret i en gruppe.*  'Emil becomes/is interested in a group.' |
| *Lise bliver på et motiveret håndboldhold.*  'Lise remains on a motivated handball team.' | *Lise bliver motiveret på et håndboldhold.*  'Lise becomes/is motivated on a handball team.' |
| *Hanne bliver i et lønnet fleksjob.*  'Hanne remains in a paid flex job.' | *Hanne bliver lønnet i et fleksjob.*  'Hanne becomes/is paid in a flex job.' |
| *Pia bliver på en omtalt bogmesse.*  'Pia remains at a mentioned book fair.' | *Pia bliver omtalt på en bogmesse.*  'Pia becomes/is mentioned at a book fair.' |
| *Maja bliver på et overspulet skibsdæk.*  'Maja remains on a sluiced ship deck.' | *Maja bliver overspulet på et skibsdæk.*  'Maja becomes/is sluiced on a ship deck.' |
| *Tina bliver til en ophidset demonstration.*  'Tina remains at an agitated demonstration.' | *Tina bliver ophidset til en demonstration.*  'Tina becomes/is agitated at a demonstration.' |
| *Jesper bliver i en opvarmet sauna.*  'Jesper remains in a heated sauna.' | *Jesper bliver opvarmet i en sauna.*  'Jesper becomes/is heated in a sauna.' |
| *Anders bliver hos en beruset veninde.*  'Anders remains with an intoxicated friend.' | *Anders bliver beruset hos en veninde.*  'Anders becomes/is intoxicated with a friend.' |
| *Ole bliver på en snavset legeplads.*  'Ole remains on a dirty playground.' | *Ole bliver snavset på en legeplads.*  'Ole becomes/is dirty on a playground.' |
| *Thomas bliver på en forfinet privatskole.*  'Thomas remains at a refined private school.' | *Thomas bliver forfinet på en privatskole.*  'Thomas becomes/is refined at a private school.' |
| *Søren bliver i en indviet kreds.*  'Søren remains in an initiated circle.' | *Søren bliver indviet i en kreds.*  'Søren becomes/is initiated in a circle.' |
| *Bente bliver i en organiseret forening.*  'Bente remains in an organized association.' | *Bente bliver organiseret i en forening.*  'Bente becomes/is organized in an association.' |
| *Karen bliver i et kontrolleret fængsel.*  'Karen remains in a controlled prison.' | *Karen bliver kontrolleret i et fængsel.*  'Karen becomes/is controlled in a prison.' |
| *Lone bliver på en overvåget arbejdsplads.*  'Lone remains in a monitored workplace.' | *Lone bliver overvåget på en arbejdsplads.*  'Lone becomes/is monitored in a workplace.' |
| *Marie bliver i en isoleret bjerglandsby.*  'Marie remains in an isolated mountain village.' | *Marie bliver isoleret i en bjerglandsby.*  'Marie becomes/is isolated in a mountain village.' |
| *Mette bliver på et overset fodboldhold.*  'Mette remains on an overlooked football team.' | *Mette bliver overset på et fodboldhold.*  'Mette becomes/is overlooked on a football team.' |
| *Henrik bliver i et styrket bofællesskab.*  'Henrik remains in a strengthened flatshare.' | *Henrik bliver styrket i et bofællesskab.*  'Henrik becomes/is strengthened in a flatshare.' |
| *Martin bliver i en etableret teatergruppe.*  'Martin remains in an established theater company.' | *Martin bliver etableret i en teatergruppe.*  'Martin becomes/is established in a theater company.' |
| *Morten bliver i et integreret team.*  'Morten remains in an integrated team.' | *Morten bliver integreret i et team.*  'Morten becomes/is integrated in a team.' |
| *Peter bliver på et skjult loftrum.*  'Peter remains in a hidden attic.' | *Peter bliver skjult på et loftrum.*  'Peters becomes/is hidden in an attic.' |
| *Emil bliver på et hjemsøgt slot.*  'Emil remains in a haunted castle.' | *Emil bliver hjemsøgt på et slot.*  'Emil becomes/is haunted in a castle.' |
